# Urethral Caruncle in Pediatrics: A Northern Tanzania Experience

**DOI:** 10.1155/2024/6104687

**Published:** 2024-04-03

**Authors:** Anteneh Tadesse Kifle, Janeth Mpelumb, Frank Bright, Orgeness Jasper Mbwambo, Tizazu Abebayehu Tsega

**Affiliations:** ^1^Department of Surgery, PCEA Chogoria Hospital, Chogoria, Kenya; ^2^Department of Urology, Kilimanjaro Christian Medical University College, Moshi, Tanzania; ^3^Department of Surgery, Hawassa University, Hawassa, Ethiopia

## Abstract

Urethral caruncles are the most frequent benign tumors of the female urethra. Most of them are found in postmenopausal women, and they are rare in childhood. Only a few pediatric cases have been published in the literature. In this report, we present a case series of three pediatric patients with a urethral caruncle.

## 1. Introduction

Urethral caruncles are benign and pedunculated and appear as sessile polypoid lesions commonly over the posterior lip of the female urethral meatus, occurring primarily in postmenopausal women, although occasionally found in premenopausal women and prepubertal girls [1, 2].

Many theories have been implicated on the etiology and pathogenesis of urethral caruncle, but none is able to explain it satisfactorily. Some of the theories include infection and chronic inflammation [3], the rupture of cysts of Skene's ducts [4], and shrinkage of vaginal tissue with secondary changes occurring due to altered environmental conditions [5].

Urethral caruncles, although usually asymptomatic and incidental findings, can cause distressing physical symptoms ranging from bleeding, pain, soreness, tenesmus and dysuria, urine flow obstruction, and significant anxiety [6]. Histologically, caruncle can be divided into three types: papillary, angiomatous, and granulomatous type [7].

The standard treatment options depend on clinical presentation. Asymptomatic patients require only reassurance and education, but there is no consensus on the optimal treatment of those with urethral prolapse who present with symptoms. The current management options for symptomatic urethral caruncle include conservative treatment and surgery [1, 6]. There are different surgical approaches to achieve these goals: electrofulguration, excision with or without cauterization, ligation, and transurethral plasmakinetic resection [6].

The purpose of this study is to report a case series of three urethral caruncles in the female pediatric age group.

## 2. Case 1

A four-year-old female child presented with pain during the initiation of micturition associated with postvoiding dribbling for three days. A spot of fresh blood was noticed on her underwear which worsened during urination and walking and decreased on resting. She had no history of frequency, straining, spraying of urine, and feeling of incomplete bladder emptying, and she can postpone voiding. No history of trauma to the perineum.

**Figure 1:**
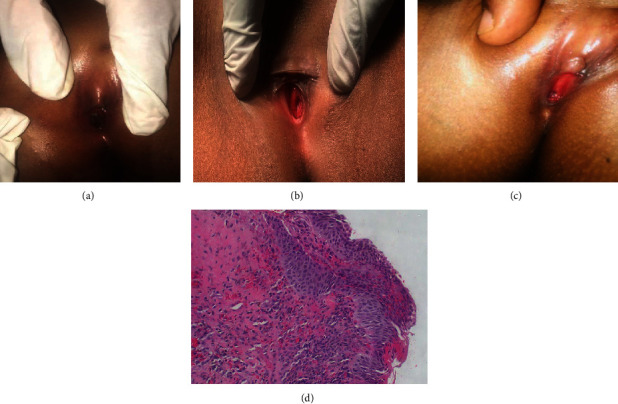
Urethral caruncle in (a) gangrenous foul-smelling mass at (b) 2^nd^ post admission day with polyploidy mass surrounding the urethral meatus which is open anteriorly and (c) 3 months postop at follow-up clinic. (d) Histology.

On physical examination, she had normal labia majora and minora. There was a 2∗1 cm necrotic mass seen on the anterior urethral meatus occluding the vaginal orifice. It was foul-smelling, fragile, and tender. An antibiotic (ceftriaxone and metronidazole) was started, and the patient was stabilized; after 48 hours, the necrotic tissue sloughed off (Figure 1(a)).

She was planned for examination under anesthesia (EUA) and cystoscopy under general anesthesia (GA). She was taken to theater, cleaned, and draped under sterile technique. Upon EUA circumferential, a reddish mass was seen at the external meatus, easily bleeding with a mild foul smell but no discharge (Figure 1(b)).

During urethrocystoscopy, there was hyperemic inflamed urethral mucosa and bladder neck. The bladder mucosa was normal, and both ureteric orifices were seen and normal.

Excision of the mass with electrocautery was done. The urethral mucosa was approximated, and tissue was sent for histopathology. A catheter was inserted and was removed after 5 days.

Hydrocortisone cream was applied after catheter removal for two weeks. There was no recurrence up to 6 months postoperatively (Figure 1(c)).

Histology results showed papillomata's lesion with vascular and fibroblastic connective tissue with chronic inflammation (Figure 1(d)), and the diagnosis of urethral caruncle was reached.

## 3. Case 2

A two-year-old child presented with a vaginal mass with on-and-off bleeding for 8 days prior to attendance at our facility. She denied a history of trauma and sexual abuse. Blood workups and abdominopelvic ultrasound scan were normal.

**Figure 2:**
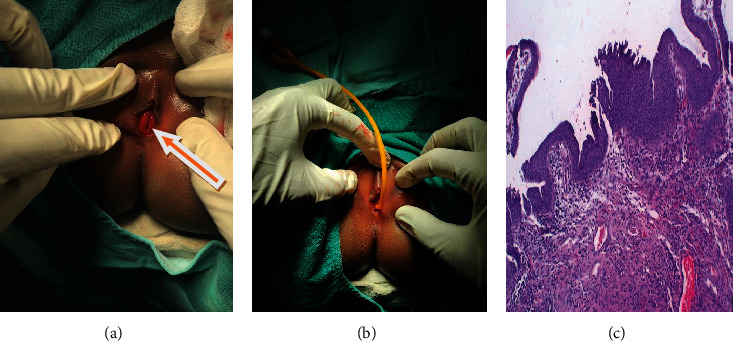
(a) Urethral caruncle, (b) postop with the catheter in situ, and (c) histology revealed.

She was then planned for urethrocystoscopy and examination under anesthesia (EUA). The EUA finding was fleshy, an outgrowth of the posterior urethral meatus was seen (Figure 2(a)), and the vagina was appreciated; catheter 8Fr passed through the assumed urethral orifice and confirmed.

Excision of the bulged part of the urethral meatus was done by electrocautery, and approximate 3 mm specimen was sent for histopathology examination. A catheter was inserted for three days (Figure 2(b)). Postoperatively, hydrocortisol cream was applied for postoperatively. The biopsy result revealed papillomata's lesion with vascular and fibroblastic connective tissue with chronic inflammation; the epithelium is hyperplastic (Figure 2(c)). Urothelial invagination with rounded nests with glandular luminal spaces and diagnosis of urethral caruncle was concluded. There was no recurrence up to 6 months postoperatively.

## 4. Case 3

A five-year-old girl presented with a complaint of blood spotting per genitalia for one week. She has no associated painful urination, frequency, or urgency. She passes clear urine. She has no history of genital trauma or sexual assault. She has a similar complaint a month back and was taken to another hospital where she was diagnosed with urethral caruncle and given estradiol topical gel for one week, and symptoms were resolved for three weeks.

**Figure 3:**
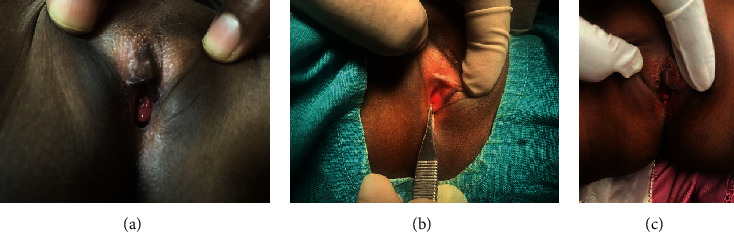
(a) Urethral caruncle in a five-year-old female. (b) Mass protruding per vagina. (c) Circumferential mass except anteriorly. Three-month follow-up with no recurrence.

On per vaginal inspection, she has a normal external genitalia appearance, with normal labia; there is a 1 cm protruding mass around the urethral meatus. It has involved the circumference of the urethra except the anterior part (Figures 3(a) and 3(b)).

She was taken to operation theater after preparation. The physical findings were confirmed. Electrosurgical excision was done. The urethral mucosa was approximated, and the catheter was left for three days. Postoperatively, hydrocortisol was applied for two weeks postoperatively. There was no recurrence for three months on follow-up (Figure 3(c)).

## 5. Discussion

In 1750, Samuel Sharp described a urethral caruncle for the first time. It is one of the most common benign lesions of the female urethra [2]. It is a benign polyploidy lesion that typically occurs in the posterior lip of the urethra meatus [8]. Mostly, it occurs in postmenopausal women [2]. There are case reports of the disease occurring in males [8, 9].

Chiba et al. reviewed the English literature during their publication of a case with literature review, who had found 14 pediatric cases of urethral caruncle. But, they were only able to review four cases including their case [1]. There are additional three cases reported in the English literature with the youngest being at three months old after their publication [2, 10, 11]. Here, we report our experience of 3 cases of pediatric urethral caruncle.

Most pediatrics' cases present with bleeding after urination or on toilet paper. It is also common to have urinary complaints like dysuria, urgency, and frequency [10]. In adults, rarely, it presents as a gigantic mass with acute or chronic urinary outflow obstruction [12]. The exact cause of urethral caruncle is unknown, but the correlation with chronic inflammation is well stated [1]. Yakasai et al. suggested that the pathogenesis also has a congenital component as it has been well documented in the literature at birth [10].

Urethral caruncle should be differentiated from other urethral lesions including urethral diverticulum, urethroceles, urethral varicosity, peri urethral gland abscess, condylomata, and cysts of Skene's gland. A number of lesions can mimic urethral caruncle: urethral melanoma, tuberculosis, lymphoma, urethral leiomyoma, urethral hamartoma, and urethral carcinoma [6].

Asymptomatic patients only need reassurance and education. Symptomatic patients will be treated conservatively or with surgical excision. The conservative approach includes warm sitz bath, estrogen creams, and anti-inflammatory drugs. This approach is often ineffective and associated with recurrence in adults. The surgical approach is the best preferred management option [13].

The principles of surgical excision of the urethral caruncle were formulated in 1926 by Ferrier and include complete eradication, restoration of the urethra meatus to normal, avoiding stricture or pulling down of the bladder neck, preserving a specimen for histology, and making the procedure simple and convenient for faster recovery [14].

There are different surgical approaches to achieve these goals: electrofulguration, excision with or without cauterization, ligation, and transurethral plasmakinetic resection. The recurrence rate after surgical treatment ranges from 0 to 12% with plasmakinetic resection having the best outcome [6]. A recurrence of urethral caruncle post surgical excision has been reported from one year to 15 years postsurgery [15]. In our case, we have done simple resection with electrocautery. Postoperatively, steroid is used to avoid recurrence after removal of the catheter and get a good outcome. There was no recurrence for all cases at six months.

## 6. Conclusion

The urethral caruncle is a rare disease in pediatric girls. Children mostly present with bleeding after urination or on toilet paper. Symptomatic patients can be treated conservatively or with surgical excision. We have used electrosurgical excision with postoperative application of steroid cream.
